# Arbuscular mycorrhizal fungi (AMF) enhance the tolerance of *Euonymus maackii* Rupr. at a moderate level of salinity

**DOI:** 10.1371/journal.pone.0231497

**Published:** 2020-04-14

**Authors:** Zhen Li, Na Wu, Sen Meng, Fei Wu, Ting Liu

**Affiliations:** 1 Institute of Applied Biotechnology, School of Life Science, Shanxi Datong University, Datong, Shanxi, China; 2 State Key Laboratory of Tree Genetics and Breeding, Research Institute of Tropical Forestry, Chinese Academy of Forestry, Guangdong, China; 3 2011 Collaborative Innovation Center of Jiangxi Typical Trees Cultivation and Utilization, College of Forestry, Jiangxi Agricultural University, Nanchang, Jiangxi, China; 4 College of Biology and Agriculture, Zunyi Normal College, Zunyi, Jiangxi, China; Consiglio per la Ricerca e la Sperimentazione in Agricoltura, ITALY

## Abstract

Salt stress is one of the major environmental constraints for plant growth. Although the ways in which mycorrhizal plants deal with salt stress have been well documented, it still is blank for *Euonymus maackii*, an important local ecological restoration tree, to arbuscular mycorrhizal fungi (AMF) inoculation and salt stress. In this study, we tested the effect of different salt levels (0, 50, 100,150 and 200 mM) and AMF inoculation on *E*. *maackii* growth rate, photosynthesis, antioxidant enzymes, nutrient absorption and salt ion distribution. The results indicated negative effect of salt on height, photosynthesis capacity, nutrition accumulation, while salt stimulated the antioxidant defense system and salt ions accumulation. The toxic symptom by excessive accumulation of salt ions worsen with salt level increased gradually (except for the 50 mM NaCl treatment). AMF inoculation alleviated the toxic symptom under moderate salt levels (100 and 150 mM) by increasing photosynthesis capacity, accelerating nutrient absorption and activating antioxidant enzyme activities under salt stress. Meanwhile, effect of AMF was not detected on seedlings under slight (0 and 50 mM) and high (200 mM) NaCl concentration. Our study indicated AMF had positive impact on *E*. *maackii* subjected to salt, which suggested potential application of AMF- *E*. *maackii* on restoration of salt ecosystems.

## 1. Introduction

Salt stress is one of the major environmental constraints for plant growth and large amounts of lands in the world are affected by excessive salt ions [1]. Datong Basin in Shanxi Province is a region with distinctive topographical and geological features. This area is characterized by salinity, with total salinity of 4–10 g/kg soil, partly explained by extreme weather and coal industrial pollution [2]. Excessive salt ions in soil will cause native plants to exhibit decreased growth height, weaker photosynthetic capacity, a disordered oxidation defense system, lower nutrient absorption and even death, eventually resulting in ecological environment deterioration [3–5]. Many techniques are employed to restore saline soils, among which the application of soil microorganisms in saline soils is considered a bio-amelioration method for plants [6].

As a native plant that is widespread in Datong Basin, *Euonymus maackii* Rupr., is important for the restoration of ecosystems [7, 8]. *E*. *maackii*, a small shrub is a species in the genus *Euonymus*, which contains approximately 145 to 183 species, and belongs to the family Celastraceae, order Celastrales [9]. It is an ideal plant for afforestation and landscaping and, has a strong ability to adapt to abiotic stress [7]. Previous studies have mainly focused on its breeding techniques, bioactive components, physiological characteristics and use in biodiesel energy [7–11]. However, the occurrence of microorganisms and *E*. *maackii* has not been reported before.

Plants exposed to soil salinity, in addition to employing some intrinsic mechanisms of adaptation, can improve their tolerance by establishing a mutualistic relationship with rhizosphere microorganisms [3,12,13]. Among soil microorganisms, arbuscular mycorrhizal fungi (AMF) have symbiotic relationships with most terrestrial plant species [14]. Previous studies have indicated that AMF can confer salt tolerance to host plants by stimulating enzyme activity protection systems, increasing photosynthesis capacity and enhancing nutrients uptake [15–16]. However, not all AMF species function equally well in improving the salt tolerance of host plants. Some researchers have reported that *Funneliformis mosseae* was the most efficient fungus for tackling salt stress [17], while others showed that for *Acacia nilotica*, herbs, woody and perennial plants, *G*. *fasciculatus* was a better choice for reducing the negative effects of salt stress [18]. The inconsistent effects of AMF inoculation could result from the salt tolerance of either the fungal species or host species [16].

For the first time, to the best of our knowledge, we attempted to investigate the effects of AMF inoculation on *E*. *maackii* exposed to salt stress and its potential ecological restoration function in the Datong Basin in this work. There were two hypothesis in our study: (1) salt stress will limit plant growth, weaken photosynthesis capacity, stimulate the antioxidant defense system and decrease nutrient contents, especially under higher salinity conditions; and (2) arbuscular mycorrhizal (AM)inoculation will enhance the salt tolerance of *E*. *maackii* by increasing photosynthesis capacity, accelerating nutrient absorption and activating antioxidant enzyme activities. With few studies about the rhizosphere microorganisms of *E*. *maackii*, especially mycorrhizas, we aimed to determine the potential function of AMF in the alleviation mechanisms that are active under salt stress and thus eventually promote acclimatization to the local ecosystem.

## 2. Materials and methods

### 2.1 Plant and soil treatments

Seeds of *E*. *maackii*, provided by the Shanxi Forestry Technology Promotion Station, were disinfected with 0.5% KMnO_4_ solution for 20 min, washed three times with distilled water and soaked in sterile water for 24 h. After that, the seeds were put on a tray covered with gauze and rapidly germinated in a 25°C light incubator. During this period, the seedlings were illuminated for 12 h every day, and the water was changed once a day. Once the seedlings had grown to 0.1 cm, they were put into a bowl (47 cm × 33 cm, 66 holes) filled with sterilized vermiculite (3 seeds/hole) and placed in a 25°C light incubator for further cultivation. The seedlings were watered every morning (20 ml/hole) for 1 month, and fully grown seedlings were transplanted to the pots descripted as following.

Topsoil (5–20 cm) was collected from an *E*. *maackii* plantation in Shanxi Province, China. The soil was sieved through a 2 mm sieve, mixed with fine washed sand at 1:1.2 (W:W) and then autoclaved at 0.11 MPa and 121°C for 2 h to supply a soil substrate for *E*. *maackii* seedlings. The soil physicochemical properties were as follows: pH value 7.9 (the ratio of soil and water was 1:5 by PHS-3B pH device), soil organic carbon (SOC, by the K_2_Cr_2_O_7_ oxidation method) 16.44 g·kg^-1^, available N (by the AA3 high resolution digital colorimeter) 33.25 mg·kg^-1^, available P (by the sodium bicarbonate extraction method and the molybdenum antimony colorimetry) 10.37 mg·kg^-1^ and available K (by the sodium bicarbonate extraction method and flame photometry) 112.17 g·kg^-1^. Compared with soil, nutrition of sand used was very few and could be ignored.

### 2.2 AMF inoculation

The inoculum AMF was *Rhizophagus intraradices* (Schenck & Smith) (BGC BJ09, Beijing Academy of Agriculture and Forestry Science), which contained spores (50 spores per gram of inoculum), mycelia, root fragments and soil.

### 2.3 Experimental design

Our experiment consisted of a randomized complete block design with two factors: AMF inoculation and salt stress (0, 50, 100, 150, and 200 mM). Salinity level were set by our preliminary experiment, and seedlings could not survive more than 1 month when subjected to 250 mM. We used 15 replications for each treatment, totaling 2 × 5 × 15 = 150 pots. The 150 seedlings were transplanted in 15 cm × 13 cm pots filled with soil substrate (2 kg) and grown in an artificial climatic greenhouse at 25°C with 12 h light per day and stable humidity of 50%. Half of the pots, were inoculated with AMF (30 g of inoculum per pot), while the remaining pots were inoculated with sterile inoculum as nonmycorrhizal controls. For the inoculation group, the inoculum was put 2 cm below the surface of the substrate, and 1 seedling was transplanted into each pot. For the control group, to kill the live inoculum, the inoculum was autoclaved with 15 ml washing solution and filtered through a 1 μm nylon mesh. After being attended to for 3 months, all pots were divided into five groups, and each group contained 15 individuals. For the salt stress groups, each group was treated with different concentrations of NaCl solution (10 mM, 20 mM, 30 mM, or 40 mM) every 2 days until reaching the final concentration, with 5 replicates. The control group was treated with sterilized water. Salt stress lasted for 1 month. According to our preliminary experiment, treatments of 100 mM and 150 mM salt level were set as moderate concentration in this study.

### 2.4 AMF colonization rate

After 1-month salt treatment, the seedlings were harvested, and the fresh roots were collected, washed, cut into 1 cm segments and fixed with FAA solution. According to the method described by Phillips and Hayman [19], 10% KOH and 0.05% trypan blue in lactophenol were used to clear and stain roots. The AM colonization rate was determined by the gridline intersection method [20]. Data were recorded as the proportion of root length colonized. AMF presence in the roots was confirmed by nested polymerase chain reaction (PCR) with SSUmAf/LSUmAr and SSUmCf/LSUmBr primers [21].

### 2.5 Growth height

To quantify the growth response, the growth in height (GH) was calculated to represent the average amount of growth over one month, determined from measurements at the beginning and end of the last 30-day NaCl treatment.

### 2.6 Gas exchange

The gas exchange parameters of the fourth and fifth fully expanded leaves, including the net photosynthesis (Pn), transpiration rate (E), stomatal conductance (Gs) and intercellular CO_2_ concentration (Ci), were determined between 8.00 and 11:30 am by a Li-Cor 6400XT portable photosynthesis measuring system (Lincoln, USA) within a week before harvest. Instrument parameters: temperature 25°C, light intensity 1000 μmol·m^-2^·s^-1^, relative humidity 50% and ambient CO_2_ 400 μmol·mol^-1^.

### 2.7 Chlorophyll fluorescence

The fourth and fifth fully expanded leaves of selected plants were placed in dark conditions (25°C, 30 min) before detecting the minimum fluorescence (Fo) and the maximal fluorescence (Fm) yields by a MINIPAM chlorophyll fluorometer (Walz, Germany). Fv/Fm represented the maximum quantum yield of photosystem II (PSII), and ΦPSII indicated the actual quantum yield of PSII. We also calculated nonphotochemical quenching (qN) and photochemical quenching (qP) by using the following formulas:

$$qN=(Fm-{Fm}^{\prime })/{Fm}^{\prime }$$

$$qP=({Fm}^{\prime }-F)/({Fm}^{\prime }-{Fo}^{\prime })$$

Fv/Fm=(Fm−Fo)/Fm

$$\Phi \mathrm{PSII}=\left(\mathrm{Fm}\prime -\mathrm{Fs}\right)/{\mathrm{Fm}}^{\prime }$$

### 2.8 Antioxidant enzyme activities

Superoxide dismutase (SOD, EC1.15.1.1) activity was assayed by the superoxide radicals generated photochemically at 560 nm, as described by Dhindsa et al. [22]. Catalase (CAT, EC1.11.1.6) activity was assayed by measuring the absorbance of the reaction mixture containing K-phosphate and H_2_O_2_ at 240 nm and peroxidase (POD, EC1.11.1.7) activity was expressed in 0.001 per minute at 470 nm following the procedure described by Mallick and Mohn [23].

### 2.9 Nutrient distribution measurement

After being dried at 80°C to a constant weight, plants were separately ground and passed through a 20 μm mesh screen. N content was measured by the semi micro-Kjeldahl method with a KjeltecTM 8400 Analyzer Unit (FOSS-Tecator, Hoganas, Sweden) [24]. Phosphorus (P) content was measured by the vanadomolybdate method [25]. K content was measured using flame atomic absorption spectrophotometry [26].

After drying at 80°C for 48 h, the plants were separately ground to a homogeneous powder and passed through a 20 μm mesh screen. Na^+^ content was detected by atomic absorption spectroscopy and Cl^-^ content was detected by a modified silver ion titration method [27].

### 2.10 Statistical analysis

The data were analyzed with SPSS 17.0 software, and two-way ANOVA was adopted to examine the effects of soil salinity, *R*. *irregularis* inoculation and their interactions in seedlings. The means were compared by Duncan’s multiple range test and HSD test (*P* < 0.05). PCA was implemented to reduce all physiological parameters to the fewest dimensions (eigenvalue > 1). For PCA, highly correlated variables were removed (only one remained) and all remaining data were standardized and computed. Two-dimensional figures were made by using SigmaPlot version 10.0 [15].

## 3. Results

### 3.1 AMF colonization rate

**Fig 1:**
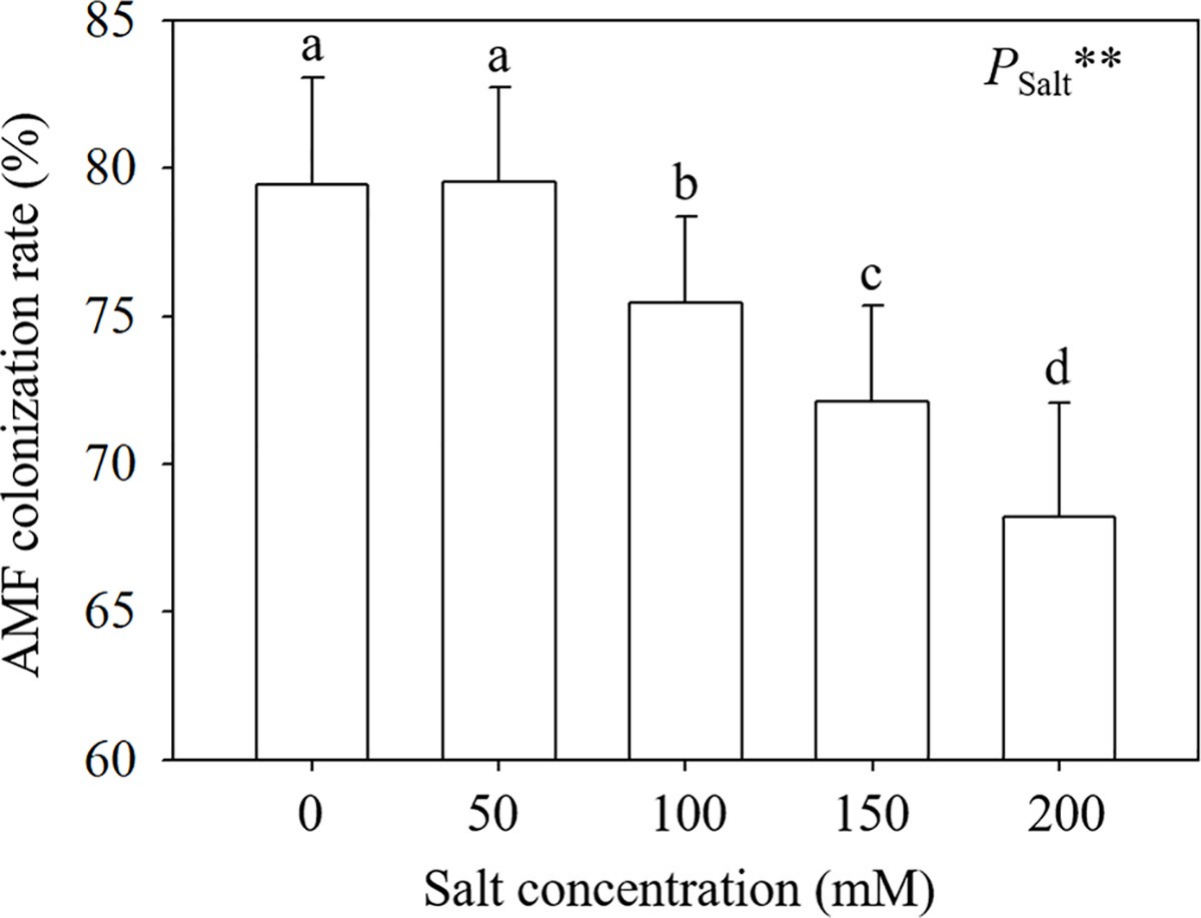
AMF colonization rate in *E*. *maackii* seedlings. **: *P* ≤ 0.01. Means with a letter in common within each variable can’t be considered different at P ≤ 0.05.

Soil salinity decreased the AMF colonization rate. The AMF colonization rates decreased from 79.44% and 79.47% (in the 0 and 50 mM treatments respectively) to 68.22% (in the 200 mM treatment) (Fig 1). And there was no significant difference between the 0 and 50 mM NaCl treatments. ANOVA results suggested the significant effects of salt levels on AMF colonization rates. Besides, no AMF inoculation was detected in samples from nonmycorrhizal treatments.

### 3.2 Height growth

**Fig 2:**
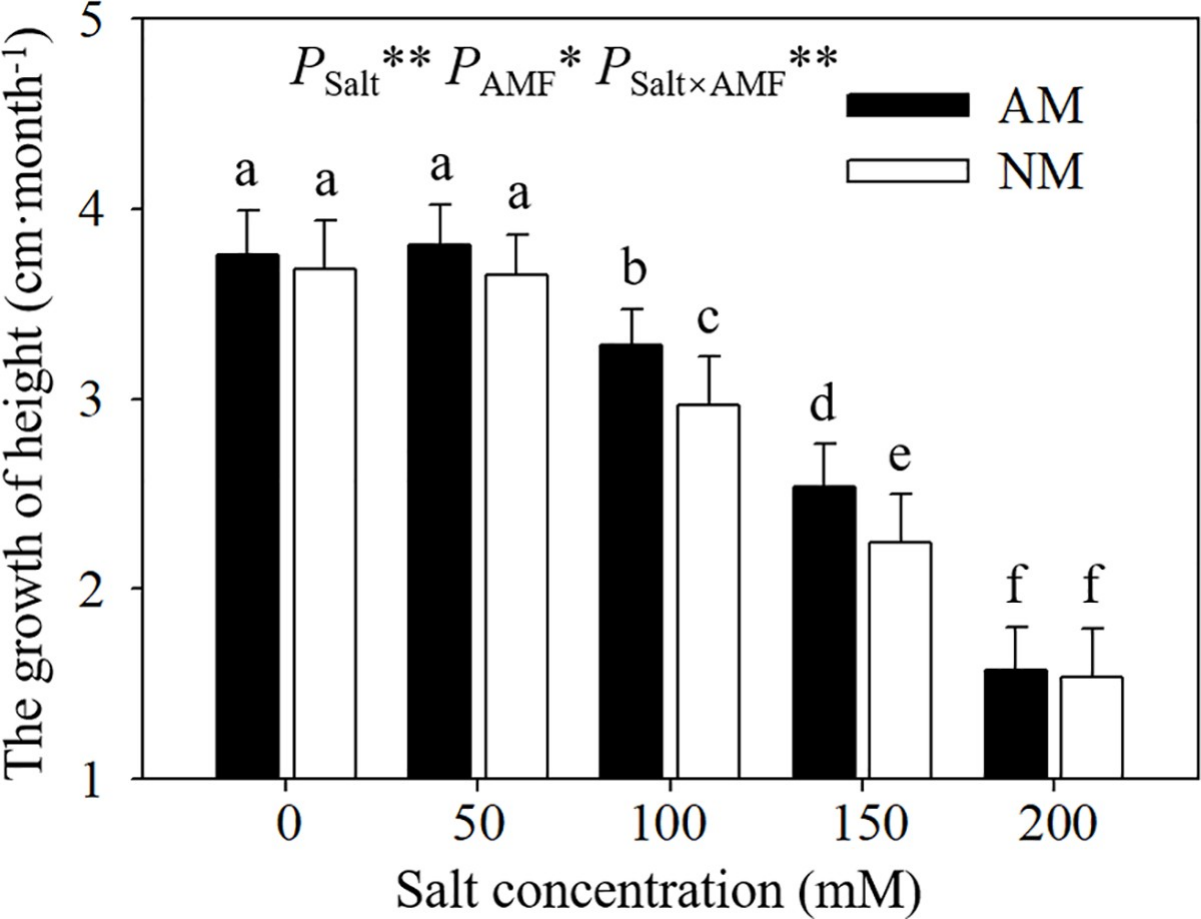
Effects of AMF inoculation on growth height of *E*. *maackii* at different NaCl levels. AM: AMF inoculation; NM: non-inoculation; AMF: AMF formation. *: significant effect at 0.01 ≤ *P* ≤ 0.05; **: *P* ≤ 0.01. Means with a letter in common within each variable can’t be considered different at P ≤ 0.05.

Salt stress caused significant declines in GH in *E*. *maackii* seedlings under NaCl stress from 50 to 200 mM. and AMF inoculation alleviated this symptom to some extend (Fig 2). The GHs of seedlings inoculated with AMF were 10.44% and 13.40% significantly higher than those of uninoculated seedlings at salinity of 100 and 150 mM. ANOVA results revealed the obvious effects by salt, AMF and their interaction.

### 3.3 Gas exchange

**Fig 3:**
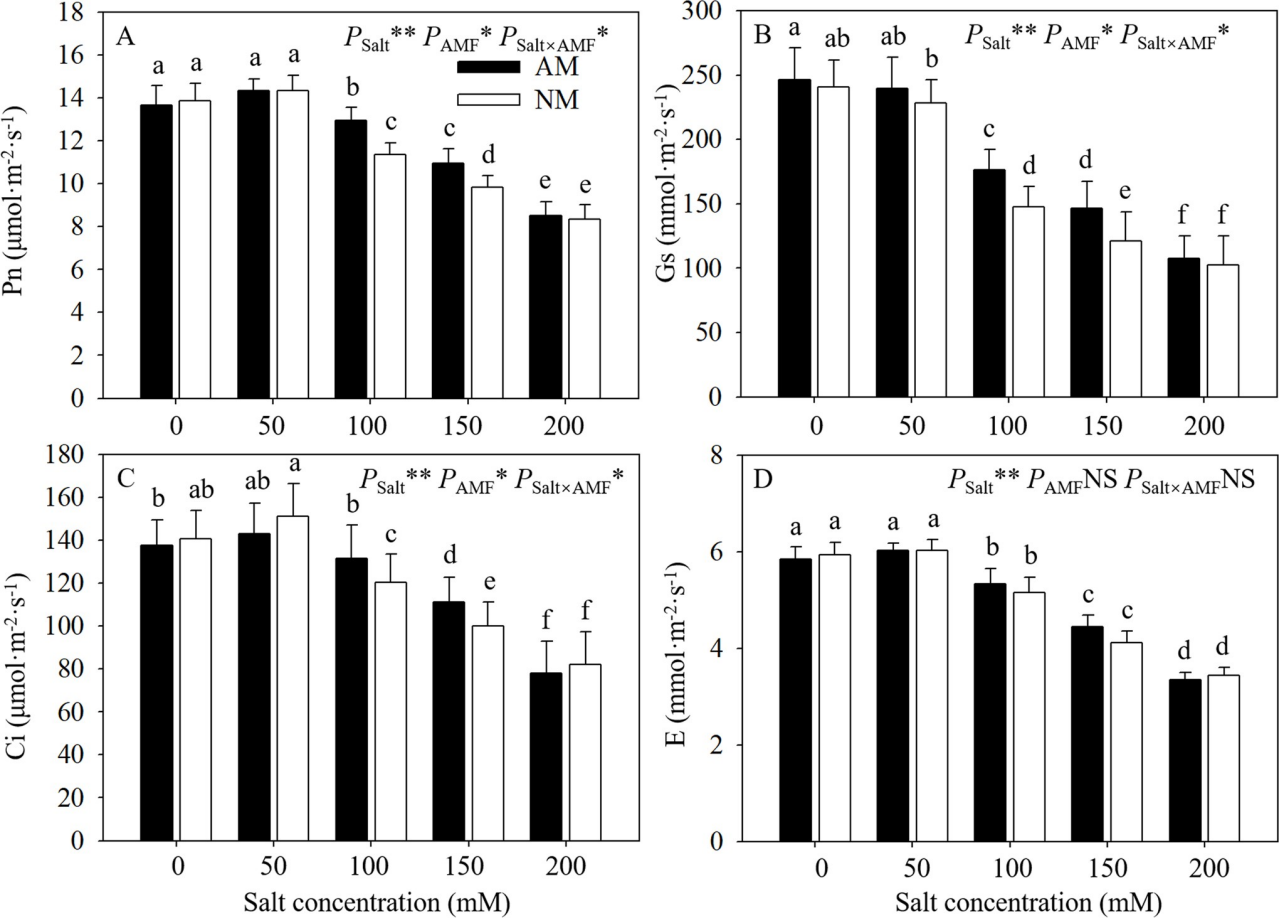
Effects of AMF inoculation on gas exchange parameters of *E*. *maackii* at different NaCl levels. AM: AMF inoculation; NM: non-inoculation; AMF: AMF formation. NS: no significant effect; *: significant effect at 0.01 ≤ *P* ≤ 0.05; **: *P* ≤ 0.01. Means with a letter in common within each variable can’t be considered different at P ≤ 0.05.

Gas exchange indexes showed similar trends as descripted on GH values. Salt stress had a negative effect on gas exchange parameters in seedlings reflected by obvious reduction at 100, 150, and 200 mM NaCl of mycorrhizal and nonmycorrhizal seedlings, and this reduction increased with the salt stress increased gradually (Fig 3). However, in mycorrhizal seedlings subjected to salt stress, the reductions of Pn, Gs and Ci were significantly lower at 100 and 200 mM salt levels compared with nonmycorrhizal seedlings. In addition, there were no significant differences in gas exchange parameters at 0, 50 and 20 mM NaCl between mycorrhizal and nonmycorrhizal seedlings. ANOVA results suggested the effect of salt levels was significant on all the gas exchange parameters. Apart from E, the other 3 indexes were significant impacted by AMF inoculation. And the interaction of AMF and salt level significantly affected Pn, Gs and Ci.

### 3.4 Chlorophyll fluorescence

**Fig 4:**
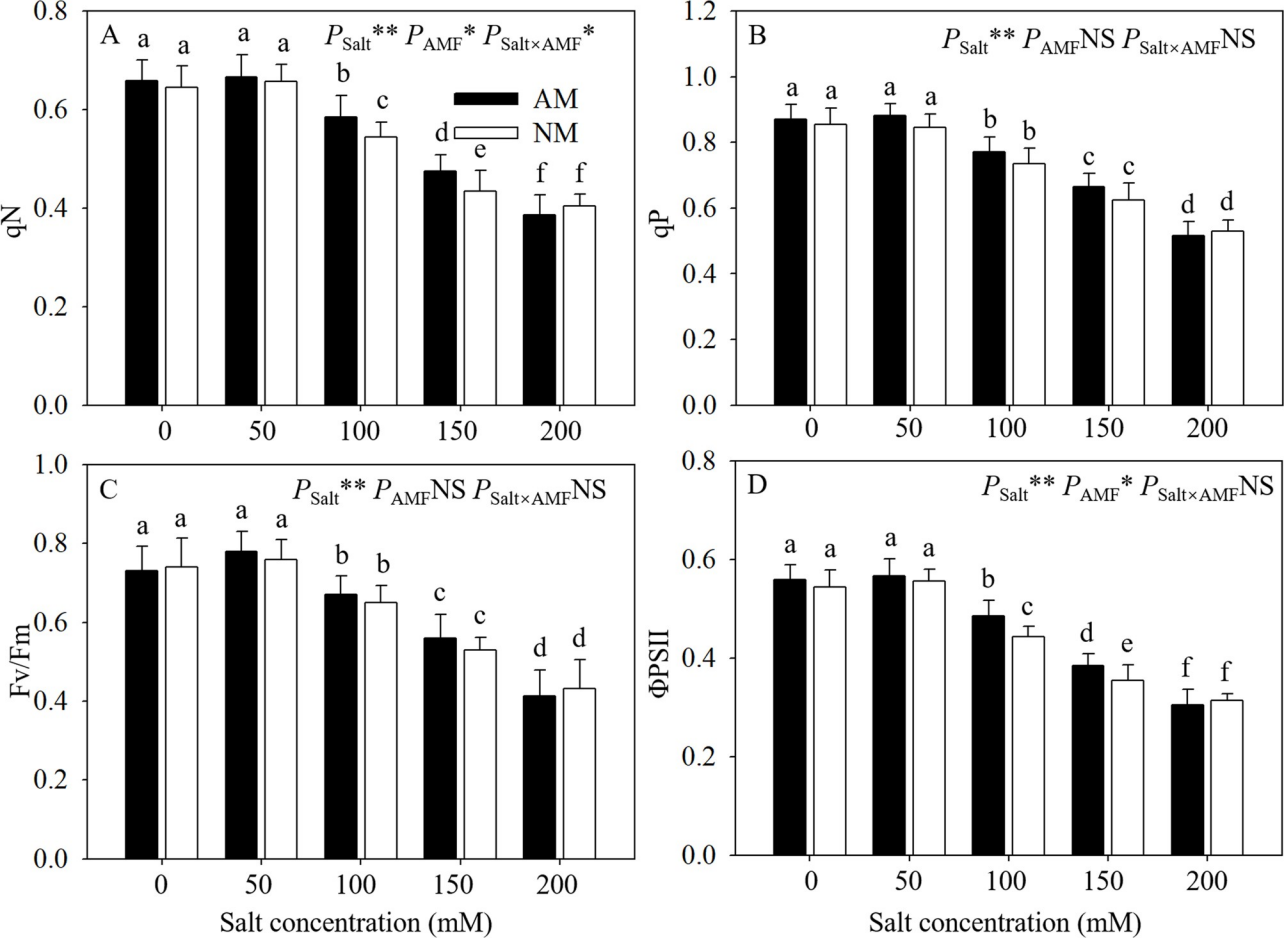
Effects of AMF inoculation on chlorophyll fluorescence parameters of *E*. *maackii* at different NaCl levels. AM: AMF inoculation; NM: non-inoculation; AMF: AMF formation. NS: no significant effect; *: significant effect at 0.01 ≤ *P* ≤ 0.05; **: *P* ≤ 0.01. Means with a letter in common within each variable can’t be considered different at P ≤ 0.05.

Salt stress significantly decreased the chlorophyll fluorescence parameters in seedlings with salt level increased gradually from 50 to 200 mM (Fig 4). Under moderate salt stress, AMF inoculation showed a positive effect on chlorophyll fluorescence parameters. Compared with nonmycorrhizal seedlings, mycorrhizal seedlings had a significantly higher qN and ΦPSII at 100 and 150 mM NaCl. Among qP and Fv/Fm, no obvious differences were detected between mycorrhizal and nonmycorrhizal seedlings under same salt level. Among qN and ΦPSII, effect of AMF inoculation was not obvious between mycorrhizal and nonmycorrhizal seedlings under 0, 50 and 200 mM respectively. ANOVA results indicated that the effect of salt levels was significant on all the chlorophyll fluorescence parameters, and the effect of AMF inoculation only significantly impacted on qN and ΦPSII. Besides, the interaction of AMF inoculation and salt level only remarkably affected qN.

### 3.5 Antioxidant enzyme activities

**Fig 5:**
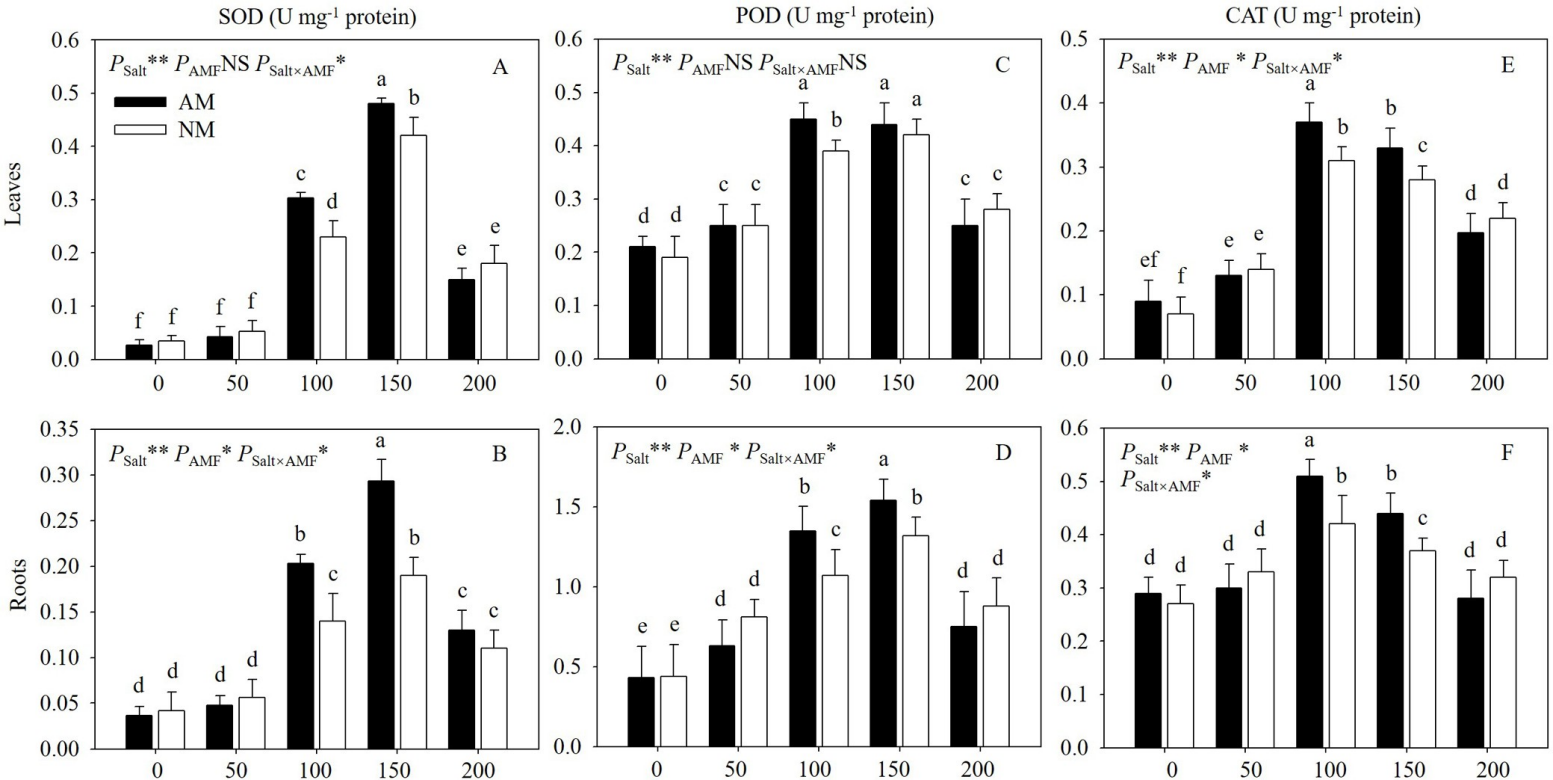
Effects of AMF inoculation on SOD, POD and CAT activities of leaves and roots of *E*. *maackii* at different NaCl levels. AM: AMF inoculation; NM: non-inoculation; AMF: AMF formation. NS: no significant effect; *: significant effect at 0.01 ≤ *P* ≤ 0.05; **: *P* ≤ 0.01. Means with a letter in common within each variable can’t be considered different at P ≤ 0.05.

With increasing salinity, the antioxidant activities first increased and then decreased, illustrating that the antioxidant system was activated by stress and was damaged under higher salt stress (Fig 5). The inoculated seedlings subjected to 100 and 150 mM NaCl showed higher SOD activity, POD and CAT activity in leaves and roots and POD activity in roots than uninoculated seedlings. However, there was no obvious difference between inoculated seedlings subjected to 0, 50 and 200 mM. Meanwhile, uninoculated plants under 50 mM showed remarkably higher enzymatic activities than those under 0 mM. Besides, no difference was detected between seedlings under 200 mM. Two-way ANOVA results showed that salt levels had significant effects on all these enzymatic activities measured, but AMF inoculation had obvious effects mainly on those of roots, and only CAT activity of three enzymes of leaves was significantly affected by AMF. This suggested the effect of AMF inoculation on the antioxidant enzyme activities were more obvious in roots than in leaves.

### 3.6 Nutrient distribution

**Fig 6:**
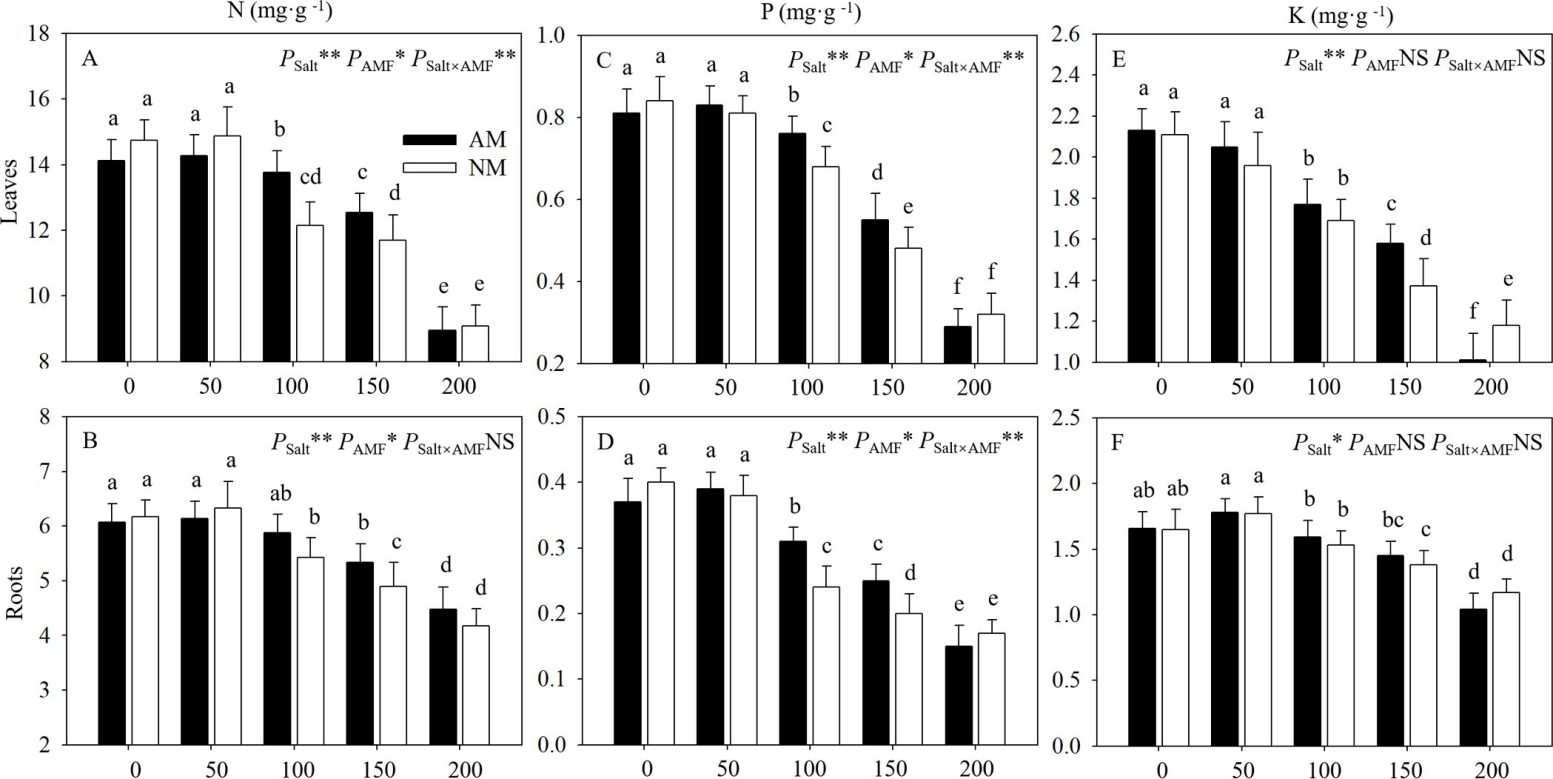
Effects of AMF inoculation on N, P and K contents of leaves and roots of *E*. *maackii* at different NaCl levels. AM: AMF inoculation; NM: non-inoculation; AMF: AMF formation. NS: no significant effect; *: significant effect at 0.01 ≤ *P* ≤ 0.05; **: *P* ≤ 0.01. Means with a letter in common within each variable can’t be considered different at P ≤ 0.05.

Gradually increasing salt levels of 50 to 200 mM lead to gradually decrease in N, P and K contents of both AMF inoculated and uninoculated seedlings (Fig 6). Compared with roots, leaves accumulated obviously more N and P, and slightly more K. N and P of leaves of inoculated seedlings were significantly higher than those of uninoculated ones at 100 and 150 mM, which was not detected under other salt levels. And among K contents this trend was only detected under 150 mM. Meanwhile, no significant differences were shown between N, P and K contents of mycorrhizal and nonmycorrhizal seedlings under 0 and 50 mM salt stress. Under 200 mM salt level, significant difference only existed in K contents of leaves between mycorrhizal and nonmycorrhizal seedlings. ANOVA results suggested that salt levels significantly affected N, P and K contents of leaves and roots, and AMF significantly affected N and P contents of leaves and roots.

### 3.7 Na^+^ and Cl^-^ determination

**Fig 7:**
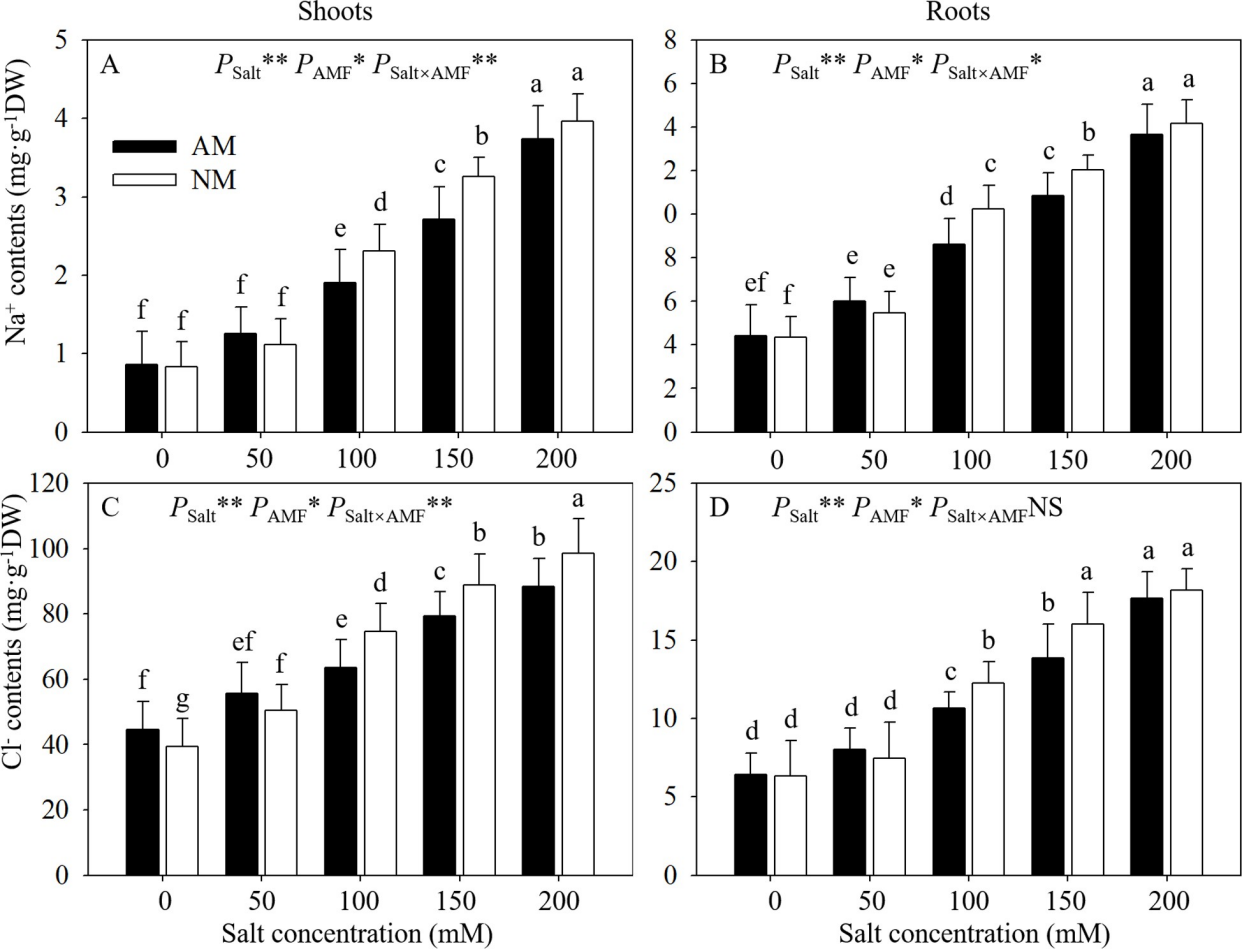
Effects of AMF inoculation on Na^+^ and Cl^-^ contents of dry matter of shoots and roots at different NaCl levels. AM: AMF inoculation; NM: non-inoculation; AMF: AMF formation. NS: no significant effect; *: significant effect at 0.01 ≤ *P* ≤ 0.05; **: *P* ≤ 0.01. Means with a letter in common within each variable can’t be considered different at P ≤ 0.05.

With salt level increasing gradually, salt ion contents in seedlings increased (Fig 7). Under salt stress, AMF inoculation significantly decreased salt ion accumulation by17.46% and 16.59% at 100 and 150 mM NaCl in leaves, while it decreased by 15.75% and 9.65% at 100 and 150 mM NaCl in roots. In addition, compared with nonmycorrhizal seedlings, mycorrhizal seedlings had significantly lower Cl^-^ contents in leaves (15.01% and 10.62% at 100 and 150 mM NaCl, respectively), and lower Cl^-^ contents in roots (13.18% and 13.50% at 100 and 150 mM NaCl, respectively).ANOVA results indicated that salt levels and AMF inoculation had significant impact on Na^+^ and Cl^-^ contents of leaves and roots, and apart from Cl^-^ contents in roots, the interactions of these 2 factors also obviously affected other parameters.

### 3.8 PCA analysis

**Fig 8:**
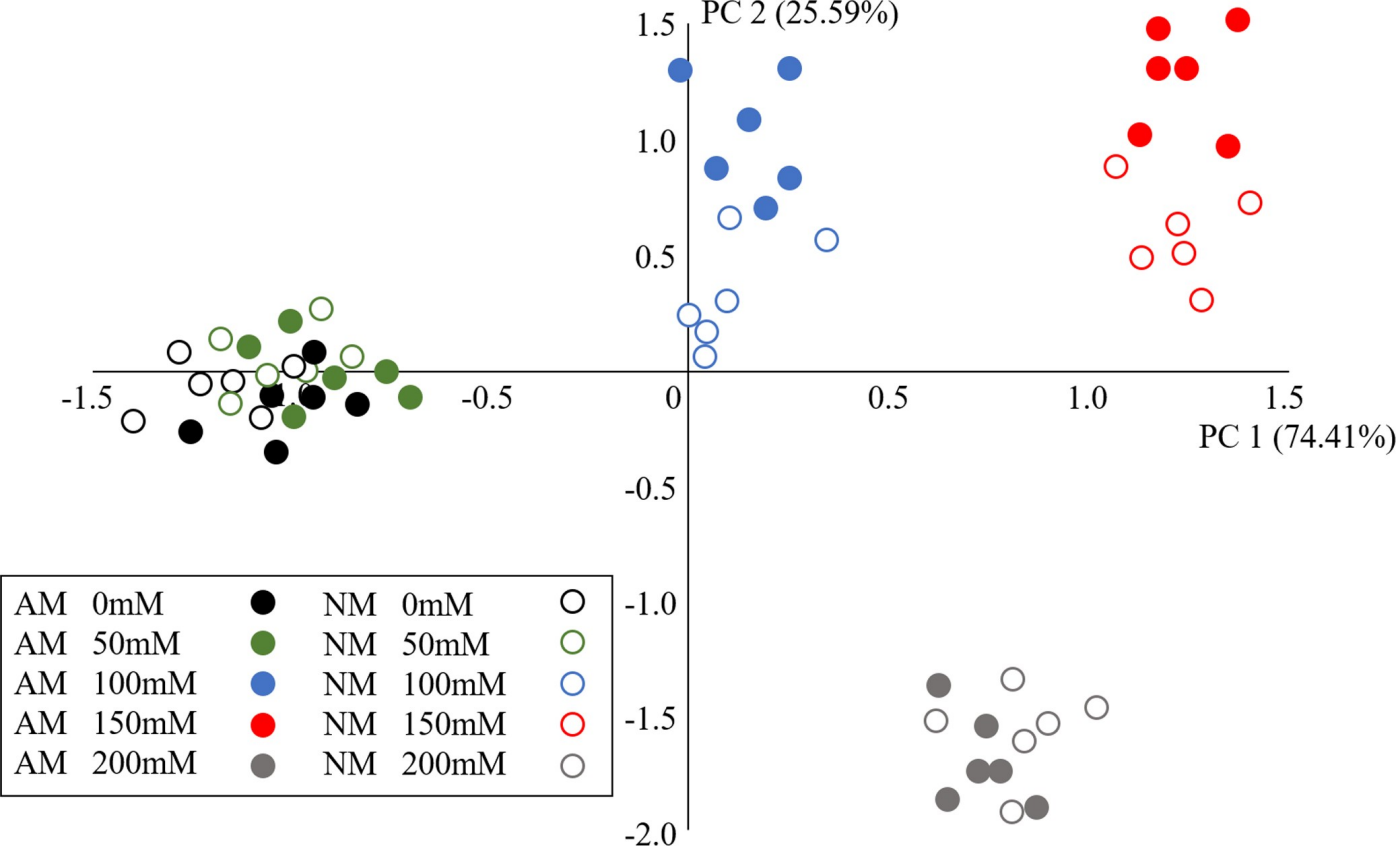
PCA plots of the two principal components of *E*. *maackii*. AM: AMF inoculation; NM: non-inoculation; PC1: principal component 1; PC2: principal component 2.

To better reveal physiological response patterns to salt stress and AMF inoculation, PCA analysis was performed by using experimental data sets. We did correlation analysis, highly correlated variables were removed: GH, Pn, Gs, Ci, E, qN, qP, Fv/Fm, ΦPSII, N content, P content, K content, Na content and Cl content were significantly correlated, SOD activities, POD activities, CAT activities were significantly correlated. We selected GH and SOD activity of leaves as presentation for following PCA process. All the physiological parameters detected in this experiment were reduced to the fewest and most representative dimensions. The distance between two treatments in the PCA figure represents the similarity of their overall status. Among all the seedlings, PC1 and PC2 accounted for 74.41% and 25.59% of the variance, respectively (Fig 8). PC1 tended to separate the salinity effects and PC2 tended to separate the AMF inoculation effect. In addition, the distances between mycorrhizal seedlings at different salt levels were closer than those of nonmycorrhizal seedlings at different salt levels (Fig 8), showing that AMF inoculation could enhance the salt tolerance of *E*. *maackii*, especially at 100 and 150 mM NaCl.

## 4. Discussion

Salt stress is a major environmental stress that drastically constrains plant growth [28]. Plants growing in saline areas are often subject to ion toxicity [1]. The toxic effects of excessive salt ions can cause great damage to the structure of enzymes, disrupt photosynthesis and induce nutrient deficiencies [3,5,15]. In this study, we tested the rate of growth in height, antioxidant enzymes, photosynthesis capacity and nutrient uptake of *E*. *maackii* exposed to salt stress and AMF inoculation to explore the potential function of AMF within *E*. *maackii* seedlings under abiotic stress.

The AMF colonization rate in *E*. *maackii* seedlings was still high (more than 68.22%) even under severe salt stress, suggesting that *E*. *maackii* is a suitable host plant for AMF. Talaat and Shawky [29] suggested that slight salt stress is beneficial to AMF colonization but excessive salt ions could weaken the AMF colonization capacity, which supported our findings that the AMF colonization of *E*. *maackii* seedlings was suppressed by salt stress from 100 to 200 mM NaCl. The early response of *E*. *maackii* seedlings to salt stress manifests as regulation of the growth rate. A severe reduction in height was observed in *E*. *maackii* when salinity stress was imposed at 150 and 200 mM NaCl. Beneficial influences of AMF on growth responses in plants under salt stress have also been found in tomato [30], poplar [15] and maize [31].

The reduction in plant growth under salt stress could be attributed to the reduction of photosynthesis capacity caused by excessive salt ions [32]. In this study, although salt stress reduced Pn in all seedlings, the Pn of mycorrhizal seedlings was still higher than that of nonmycorrhizal ones under moderate salt stress, which could result from the improved growth status of AM seedlings under salt stress. Chaves et al. [33] found that salt stress could affect CO_2_ diffusion through a decrease in mesophyll and stomatal conductance; thus, the Pn enhancement in mycorrhizal seedlings maybe related to the higher Gs than that found in nonmycorrhizal seedlings. In our study, we also observed that the same results under moderate salt stress (100 and 150 mM) and similar Gs values between different treatments at 200 mM salt. The lower reduction of Gs in mycorrhizal seedlings than nonmycorrhizal seedlings under moderate salt stress suggested that the growth status may be better in mycorrhizal seedlings, resulting from less of a decrease in CO_2_ diffusion through the stomata [34]. The Ci values were higher than those under unstressed conditions, which may indicate that enzymes in the photosynthesis apparatus were destroyed, resulting in a decrease in CO_2_ assimilation and the accumulation of CO_2_ in intercellular areas [35]. In our research, mycorrhizal seedlings had a significant higher Ci than nonmycorrhizal seedlings at 100 and 150 mM NaCl. Compared with nonmycorrhizal seedlings, mycorrhizal seedlings had a lower metabolic limit of CO_2_ assimilation [36].

Photochemical reactions represent the photosynthetic capacity of plants and are sensitive to soil salinity [37]. Many researchers have found that salt-tolerant plants are better able to maintain PSII activity than salt-sensitive plants [1]. We found that the ΦPSII of mycorrhizal seedlings were significantly higher than those of nonmycorrhizal seedlings under salt stress at 100 and 150 mM NaCl. This result may imply that mycorrhizal seedlings suffered less disorder in the electron transport chain than nonmycorrhizal seedlings [38]. In addition, AMF inoculation also increased qN significantly under moderate salt stress, and we thought AMF inoculation might increase the utilization of absorbed light and minimize the dissipation of light energy [39]. Thus, due to their higher PSII efficiency than that of nonmycorrhizal seedlings, mycorrhizal seedlings were more tolerant of moderate salt stress (100 and 150 mM NaCl).

When plants exposed to salt stress, reactive oxygen species, such as H_2_O_2_ and O_2_^-^ accumulated, which created the cytotoxic condition. To minimize the oxidative damage, the plants exposed to salt stress is always associated with the induction of antioxidant defense enzymes [40]. On general, AM formation contributed to scavenging peroxyl radials production, buffering cellular radical potential, and a more powerful reactive oxygen species scavenging system [41]. Seedlings formed AM symbiosis have been shown to have lower lipid peroxidation and higher antioxidant enzymatic activities, which supports our findings that under moderate salt stress (100 and 150 mM), the SOD and CAT activities were significantly higher in mycorrhizal leaves and roots than those of nonmycorrhizal ones. This result also illustrated the potential role of AM symbiosis in activating antioxidant enzyme activities as a protective strategy as described by Wu et al. [15]. However, there was no difference between inoculated seedlings subjected to 0 and 50 mM, which suggested the role of AMF performed better when host plants were suffering moderate stress. Besides, according to ANOVA results, we detected that AM fungi had more significant impact on the roots than the leaves. Given that the ecological niche of AMF is in the root and rhizosphere, we hypothesized that the main effects of AM symbiosis is on the roots rather than leaves.

AM symbiosis improved the absorption of nutrients, which made AMF an essential tool for plants exposed to abiotic stress [16]. Our research showed that the enhanced nutrient absorption observed with AMF inoculation was manifested in the N, P and K contents. Many researchers hold the opinion that the improvement of plant P acquisition is the most important mechanism in mycorrhizal plants under salt stress [42], such as Frosi et al. [43] found that P can be transported into the root via protoplasmic circulation, greatly reducing the transport resistance and increasing the transport speed for the continuous mycelium of AMF. However, some other studies found that even if mycorrhizal and nonmycorrhizal plants have a similar P status under unstressed conditions, mycorrhizal plants still grow better than nonmycorrhizal plants under salt stress [44]. In addition, the AMF inoculation can also promote N assimilation and absorption to reduce the toxic effects [45], which was in line with our findings on N. Different from N and P, K was an important osmotic adjustment element. Previous studies have shown that excessive accumulation of salt ions affects the selective absorption of K by interfering with related proteins, and AMF has a buffering effect on the content of Na, with a certain limit, to promote the absorption of K [34,46]. Our results showed that the nutrient contents increased significantly in mycorrhizal plants under moderate salt stress compared with those of nonmycorrhizal plants, suggesting that AMF was necessary for the plants to reach their optimal mineral nutrition under abiotic stress [47]. The enhanced nutrient absorption capacity observed may result from the fact that AMF hyphae usually penetrate deeper into the soil beyond the rhizosphere, where they can absorb nutrients under more various kinds of conditions than just at the root surface [6]. Thus, mycorrhizal seedlings could maintain photosynthesis and activate the antioxidant defense system by regulating nutrient uptake to relieve toxic effects, which supported our findings [48]. Most terrestrial plans can form exchange associations with AMF, and this exchange is a key factor in the cycling of nutrients during ecological, evolutionary and physiological processes for plants, eventually forming an important component of sustainable soil-plant systems [6,48,49].

The primary impact of excessive Na^+^ and Cl^-^ concentrations is the disturbance of photosynthesis, defense systems and nutrient uptake, leading to metabolic activity attenuation. To better survive under salt stress, it is essential for most plants to maintain lower Na^+^ and Cl^-^ contents [1,15,34]. In our study, the Na^+^ contents in seedling shoots were significantly lower than those in roots, indicating the efficient inhibition of Na^+^ transport. However, the variation in Cl^−^ content differed from that in Na^+^ content because of the opposite uptake patterns: seedlings accumulated more Cl^-^ and less Na^+^ in leaves than roots. In addition to the intrinsic mechanisms of host plants, AM symbiosis can provide external assistance under abiotic stresses [50].

Salt stress can inhibit plant growth through several mechanisms including inhibition of photosynthesis, reduced in nutrient uptake, and damage to enzymes [50,51]. In this experiment, the deleterious effects of NaCl stress could be mediated by mycorrhizal colonization at 100 and 150 mM NaCl levels. Combined with above results on colonization rate, height, photosynthesis capacity and nutrient content, AMF performed under moderate salt stress. However, as we showed, salt stress at 50 and 200 mM had no significant effect on plants, and AM inoculation had no effect. These statements above suggested that AMF performed well when host plants felt stress and the stress level wasn’t fatal. Besides, under moderate salt stress, AMF colonization rates were lower than those under 0 and 50 mM NaCl, but more efficient AM effect was detected under moderate salt stress, which suggested AM effect was not in line with its inoculation rate.

Consistent with previous reports, AMF could enhance the salt tolerance of *E*. *maackii* seedlings, but the beneficial effect was finite within the range of salt stress conditions considered. Higher salt stress can cause severe damage to both the host plant and AMF, so AMF inoculation was not effective at 200 mM NaCl. Because of the enhanced nutrient status and photosynthesis capacity, the mycorrhizal seedlings could maintain detoxifying activity by activating antioxidant enzymes under moderate salt stress.

## 5. Conclusion

Highly consistent with our hypothesis, after germinated and grew under unstressed condition, salt stress caused negative physiological effects, including limited growth, decreased photosynthesis, stimulation of the antioxidant enzyme system and lessened nutrient uptake. In addition, mycorrhizal association improved the salt tolerance of *E*. *maackii* by increasing photosynthesis, activating the antioxidant enzyme system and enhancing nutrient uptake, particularly at 100 and 150 mM NaCl. With the help of AMF, *E*. *maackii* grown under salt stress could acquire tolerance, which suggested a potential possibility of application of AMF for ecological stability in saline areas.

## Supporting information

S1 Data (XLS)
